# From test result to surveillance record: A narrative review of malaria RDT data quality in African health information systems

**DOI:** 10.1016/j.dialog.2026.100362

**Published:** 2026-10-06

**Authors:** Abdullahi Hassan Elmi

**Affiliations:** a Department of Nursing and midwifery, Dr. Sumait Hospital, SIMAD University, Mogadishu, Somalia; b Faculty of Medicine and Health Sciences, SIMAD University, Mogadishu, Somalia

**Keywords:** Malaria, Rapid diagnostic test, Routine health information system, Data quality, Surveillance, DHIS2, Africa

## Abstract

**Purpose:**

Malaria rapid diagnostic tests (RDTs) generate information used for clinical care and routine surveillance. However, surveillance reliability depends not only on whether a test correctly identifies infection but also on whether the observed result is recorded, aggregated, transmitted, and interpreted accurately. This review aimed to examine weaknesses in the pathway from malaria RDT results to routine surveillance data and identify practical approaches to improving data quality.

**Methods:**

A structured narrative search of PubMed, Google Scholar, and World Health Organization resources was conducted for English-language publications issued from January 2010 to 10 September 2026. Search terms combined “malaria,” “rapid diagnostic test,” “routine health information system,” “health management information system,” “HMIS,” “DHIS2,” “data quality,” “recording,” “reporting,” “completeness,” “concordance,” and “timeliness.” Empirical studies from African settings were prioritized. Non-African evidence was included only when it offered potentially transferable implementation lessons. Findings were synthesized according to the stages of the data pathway and the dimensions of data quality examined.

**Results:**

Evidence from Kenya, Senegal, Uganda, and a multicountry African evaluation identified incomplete reporting, discordance between source registers and DHIS2, aggregation errors, ambiguous zero and missing values, inconsistent indicator definitions, and limited feedback to healthcare facilities. Missing counts of people tested remove the denominator needed to interpret test positivity and can make changes in testing practices appear to be changes in malaria transmission. Standardized indicators, routine source verification, supportive supervision, and interoperable offline-capable digital tools may improve data quality, although their effects vary by setting. Automated RDT readers are promising but require further evaluation of feasibility, equity, governance, and sustainability.

**Conclusion:**

Malaria surveillance depends on both the validity of the original diagnostic result and the integrity of the subsequent information pathway. Data-quality interventions should be selected according to the point of failure, supported by local evidence, and evaluated before large-scale implementation.

## Highlights

• Malaria surveillance can be distorted after an RDT result has been generated.
• Missing testing denominators and source-to-DHIS2 discordance bias programme indicators.
• Standardized, interoperable systems and supportive verification may improve data quality.

## 1 Introduction

Malaria remains a major public health burden. The World Health Organization (WHO) estimated that 263 million malaria cases and 597,000 deaths occurred globally in 2023, with the WHO African Region accounting for approximately 94% of cases and 95% of deaths [1]. Effective malaria control depends on surveillance systems capable of detecting changes in transmission, directing commodities and personnel, and measuring programme performance [1], [2]. The expansion of parasitological confirmation has made information generated by malaria RDTs an increasingly important component of routine surveillance, particularly in settings where microscopy is unavailable.

Two distinct forms of reliability must nevertheless be considered. Diagnostic accuracy concerns whether an RDT correctly identifies malaria infection. Surveillance-data quality concerns whether the observed result is recorded, aggregated, transmitted, and used without distortion [3], [4], [5], [6]. A diagnostically correct result can still become an inaccurate surveillance record if it is documented or reported incorrectly. Conversely, accurate reporting cannot correct a false diagnostic result [3], [4], [5], [6].

This review therefore focuses principally on the post-test information pathway while briefly considering upstream biological, environmental, and procedural factors that determine the validity of the result entering that pathway. Its objective is to synthesize evidence on the principal points at which malaria RDT information may be lost or altered between testing and routine health information systems, explain the epidemiological consequences of these failures, and identify evidence-supported and emerging strategies for improvement.

## 2 Methods

A structured narrative review was conducted. PubMed and Google Scholar were searched, and the websites and technical resources of WHO were examined for literature published between 1 January 2010 and 10 September 2026. The search combined terms related to malaria and RDTs with “routine health information system,” “health management information system,” “HMIS,” “DHIS2,” “data quality,” “recording,” “reporting,” “completeness,” “timeliness,” “consistency,” “concordance,” “supervision,” “digital reporting,” and “automated reader.” Reference lists of relevant publications were also examined.

Eligible materials included empirical studies and authoritative technical guidance addressing at least one part of the malaria test-to-surveillance pathway. Priority was given to studies from African countries that evaluated source registers, facility summaries, routine reporting, DHIS2, or the implementation of data-quality interventions.

Evidence from outside Africa was retained only when it described a relevant information-system function or implementation lesson not adequately represented in the African studies. Its geographical origin is explicitly identified in the manuscript. Commentaries without a transparent evidentiary basis were not used to support quantitative claims.

Because the included studies differed considerably in their designs, indicators, settings, and definitions of data quality, a meta-analysis was considered inappropriate. Evidence was synthesized narratively according to the stage of the data pathway, the dimension of data quality assessed, and the implications for implementation. This approach was intended to clarify concepts and identify potentially transferable lessons rather than calculate a pooled intervention effect.

## 3 Conceptual framework for malaria RDT data quality

The term “data quality” comprises several related but distinct dimensions. Clearly defining these dimensions is important because each type of problem arises at a different point in the testing and reporting pathway and may require a different intervention. The dimensions used to distinguish diagnostic performance from post-test surveillance-data quality are summarized in Table 1.

**Table 1:** Conceptual framework for malaria RDT data quality.

Dimension	Operational definition
Diagnostic accuracy	Whether the RDT correctly classifies malaria infection relative to an appropriate reference standard
Recording accuracy	Whether the observed RDT result and associated patient information are entered correctly in the source document
Aggregation accuracy	Whether source-record values are correctly summarized in facility totals and monthly reports
Completeness	Whether all expected fields, encounters, facilities, and reporting periods are represented
Timeliness	Whether required reports are submitted within the specified reporting period
Concordance	The level of agreement among source registers, monthly summaries, and DHIS2 or another national reporting platform
Data-use quality	Whether data are reviewed, interpreted, communicated, and used to support appropriate action

The framework distinguishes the diagnostic validity of the original RDT result from the quality of its subsequent recording, aggregation, reporting, and use. The definitions were adapted from the WHO Data Quality Review framework and applied to the malaria RDT-to-surveillance pathway [3].

Abbreviations: DHIS2, District Health Information Software 2; RDT, rapid diagnostic test.

## 4 Where the RDT-to-surveillance pathway can fail

### 4.1 Validity of the result entering the system

Before documentation begins, the RDT result may be affected by parasite biology, storage conditions, test execution, or interpretation. Deletions of the *pfhrp2* and *pfhrp3* genes can cause false-negative results with HRP2-based RDTs. WHO guidance also emphasizes product selection, temperature stability, transport, storage, lot testing, correct reading time, and adherence to the manufacturer's instructions [4], [5].

Exposure to excessive heat, damaged or expired kits, incorrect blood or buffer volume, premature or delayed interpretation, and difficulty recognizing faint test lines can alter the result. These are diagnostic-performance and quality-assurance problems rather than reporting errors. Nevertheless, they determine the validity of the information subsequently entered into the surveillance system [4], [5].

### 4.2 Recording at the point of care

After an RDT has been interpreted, the result may be omitted, entered in the wrong column, linked to the wrong patient, or recorded inconsistently across outpatient, laboratory, and malaria registers. Heavy workloads, multiple parallel registers, unclear reporting responsibilities, staff turnover, and limited familiarity with indicator definitions increase the likelihood of these errors [5], [6].

An RDT cassette provides a temporary visual result. If that result is not captured accurately at the point of care, later verification may be difficult or impossible. Recording systems should therefore enable healthcare workers to document the result immediately and consistently using clearly defined fields and patient identifiers [5], [6].

### 4.3 Aggregation and transfer to DHIS2

Most aggregate reporting systems require healthcare workers or information officers to count register entries, transfer the totals to facility summary forms, and then enter those totals into DHIS2. Each transfer creates an opportunity for omission, duplication, arithmetic error, or transcription error [7], [8].

In eastern Uganda, Nuwasiima and colleagues compared selected malaria indicators recorded in DHIS2 with the corresponding facility source documents. They identified low concordance for several indicators and reported that workload, documentation practices, supervision, and health-system factors influenced reporting performance [8].

A subsequent multicountry evaluation conducted in four sub-Saharan African countries similarly demonstrated that agreement could change across facility registers, monthly summary forms, and health management information systems [9]. These findings support a pathway-specific interpretation of data quality: the same facility may perform well at one transfer point but introduce errors at another.

### 4.4 Completeness, timeliness, and representativeness

The adoption of DHIS2 does not automatically guarantee complete, timely, or representative reporting. In Senegal, facility reporting completeness increased from 84.5% in 2014 to 97.5% in 2017. Nevertheless, only 15.3% of private facilities used the reporting system, compared with 92.7% of public facilities [7]. Across the three malaria indicators examined, missing values remained an important problem, and hospitals performed less consistently than health posts and health centres [7]. In Kenya, an assessment of 19 malaria indicators reported through DHIS2 between 2011 and 2015 also documented improvements alongside persistent geographical and indicator-specific gaps [10]. These findings demonstrate why a high national reporting percentage may coexist with substantial weaknesses involving particular facility types, ownership categories, geographical locations, or indicators.

Ambiguous use of blank fields and zero values represents another important surveillance risk. A blank entry may indicate that no event occurred, the service was unavailable, the event was not counted, or the facility failed to report. Treating all blank fields as zero can underestimate malaria burden, whereas treating true zeroes as missing values can exaggerate reporting incompleteness. Explicit zero-reporting rules and automated validation should therefore be incorporated into national indicator specifications [3], [7].

## 5 Why missing denominators matter

Reporting positive RDT results without reporting the total number of people tested removes the denominator required to calculate test positivity. Test positivity is generally calculated by dividing the number of positive tests by the total number of valid tests performed. If negative tests are omitted, test positivity may be artificially inflated or cannot be calculated reliably [2], [3], [7]. If the number of patients tested changes over time, a change in confirmed cases may reflect a change in transmission, access to diagnostic services, clinicians' testing practices, or reporting completeness. Positive case counts alone cannot distinguish among these possibilities. Denominator errors also bias comparisons among facilities and districts. A facility that tests a broad group of febrile patients may report lower positivity than a facility that tests only patients with a strong clinical suspicion of malaria, even if the underlying level of transmission is similar [2], [3], [7].

Missing testing totals can consequently distort assessments of:

• Malaria test positivity.
• Diagnostic testing coverage.
• Confirmed malaria incidence.
• Geographical and temporal trends.
• Outbreak signals.
• RDT and antimalarial commodity requirements.
• Programme performance.

Surveillance systems should retain a linked set of indicators covering suspected cases, patients tested, positive results, and patients treated. These indicators should use harmonized definitions and be subjected to routine internal consistency checks [2], [3], [7].

## 6 Evidence-supported strategies

### 6.1 Indicator standardization and source verification

National malaria programmes should define each indicator, its source document, calculation method, required disaggregation, reporting frequency, and treatment of zero and missing values. Facility summaries should be reconciled with source registers before submission [3].

Selected indicators should also be verified periodically against source documents. WHO's Data Quality Review framework provides standardized approaches for assessing completeness, internal consistency, and external consistency [3].

Verification should be risk-based, prioritizing facilities or indicators with:

• Abrupt or unexplained changes.
• Repeated missing values.
• Implausible relationships between indicators.
• Persistent late reporting.
• Large differences between registers, summaries, and DHIS2.
• Unusual patterns of zero reporting.

### 6.2 Training, supportive supervision, and feedback

Training is most useful when it addresses the specific tasks that generate errors, including correct register completion, indicator calculation, zero reporting, data reconciliation, and interpretation [11].

In Kayunga District, Uganda, an intervention combining in-service training with a collaborative improvement strategy substantially improved malaria surveillance completeness but had limited effects on accuracy [11]. This finding indicates that training alone may not resolve structural weaknesses in reporting systems.

Supportive supervision should therefore include direct observation, source-document verification, joint problem-solving, and timely feedback rather than checklist completion alone. Supervisory visits should help healthcare workers identify why discrepancies occurred and develop practical corrective actions [11].

Dashboards and feedback reports are more likely to improve data use when frontline staff can investigate discrepancies and see how their data influence local decisions, including commodity distribution, outbreak response, staffing, and community intervention [10], [11].

### 6.3 Digital data capture and interoperability

Digital entry close to the point of testing can reduce repeated transcription and enable validation rules, timestamps, prompts, and faster aggregation. However, digitalization can also reproduce poorly defined indicators or create additional parallel reporting systems [12].

Digital reporting tools should:

• Function offline and synchronize when connectivity becomes available.
• Use standardized national indicators and facility identifiers.
• Exchange data with DHIS2 or the national HMIS.
• Prevent or flag duplicate records.
• Include validation and consistency checks.
• Maintain audit trails.
• Provide a documented procedure for correcting inaccurate records.
• Apply role-based access and appropriate data protection.
• Minimize unnecessary collection of patient-identifiable information.

Evidence from the Solomon Islands demonstrated that a DHIS2-supported malaria reporting system could be evaluated systematically [12]. However, this evidence originated outside Africa and should be viewed as contextual rather than directly generalizable.

Implementation in African settings must account for unreliable electricity, device replacement, internet availability, language, digital literacy, technical support, recurrent hosting or licensing expenses, and continued operation during system outages. Electronic community health information systems may reduce delays and data loss when malaria testing is performed by community health workers. Where such systems are introduced, community-level RDT records should use the same indicator definitions, patient or encounter identifiers, validation rules, and reporting periods as facility-based systems. Electronic community registers should also be interoperable with the national HMIS and capable of functioning offline. However, further comparative evidence is needed to determine whether these systems improve accuracy and completeness beyond the improvements achieved in reporting timeliness [11], [12], [13], [14].

### 6.4 Automated and AI-assisted RDT readers

Automated readers can photograph an RDT, assist with result interpretation, preserve a digital record, and link the result with time and location. A feasibility study conducted in health facilities in the Democratic Republic of the Congo described practical challenges and lessons arising from the deployment of automated RDT readers [13].

A 2025 study conducted in five health facilities in Kisumu County, Kenya, also evaluated AI-assisted RDT interpretation within a connected diagnostic system [14]. These technologies may be particularly useful for identifying faint test lines, supporting remote quality assurance, and reducing manual data transcription. Nevertheless, the current evidence is insufficient to describe automated or AI-assisted readers as universally proven solutions. Their evaluation should include:

• Diagnostic accuracy and reliability.
• Acceptability among healthcare workers and patients.
• Feasibility within existing clinical workflows.
• Algorithmic bias across RDT products and populations.
• Offline functionality.
• Interoperability with national information systems.
• Privacy and data governance.
• Cost and maintenance requirements.
• Equity of implementation.
• Sustainability after pilot funding ends.

## 7 Implementation priorities

Based on the reviewed evidence, the principal operational priorities and implementation considerations are summarized in Table 2.

**Table 2:** Implementation priorities for strengthening malaria RDT data quality and routine surveillance.

Priority	Operational action	Responsible level	Implementation outcome and evidence status	References
Indicator governance	Maintain a national indicator dictionary with explicit denominator and zero-reporting rules	Ministry of Health, national malaria programme and HMIS unit	Supports completeness, consistency and concordance	[2], [3], [7]
Source verification	Conduct routine register-summary-DHIS2 reconciliation and risk-based audits	Facility teams and district managers	Evidence-supported strategy for improving accuracy and concordance	[3], [8], [9]
Workforce support	Provide task-specific training, mentorship, feedback and induction for new staff	District and facility managers	Requires assessment of acceptability, fidelity and sustainability	[8], [11]
Digital reporting	Use offline-first tools with validation, interoperability, audit trails and correction procedures	Ministry of Health, HMIS unit and technology partners	Requires evaluation of feasibility, timeliness, privacy and total cost	[7], [10], [12]
Automated RDT reading	Introduce through context-specific pilots with independent performance and implementation evaluation	Malaria programmes, regulators and researchers	Promising approach; effectiveness and equity require further study	[13], [14]
Local data use	Link facility dashboards to review meetings and documented corrective actions	Facility and district teams	May strengthen data-use quality and accountability	[3], [7], [8], [11]

The table separates established data-quality practices from emerging digital interventions. “Evidence-supported” indicates support from observational, implementation, or technical-guidance evidence and does not necessarily indicate effectiveness established through randomized trials. Emerging interventions should be evaluated for acceptability, feasibility, fidelity, sustainability, equity, interoperability, privacy, infrastructure requirements, and cost before widespread implementation.

Abbreviations: DHIS2, District Health Information Software 2; HMIS, health management information system; RDT, rapid diagnostic test.

## 8 Research and policy priorities

Future research should identify the exact point in the information pathway at which an outcome is measured. Studies should avoid using “data accuracy” as a single undifferentiated endpoint. They should instead provide clear definitions and denominators for completeness, timeliness, concordance, and error rates [14].

Comparative evaluations are required for:

• Paper, hybrid, and fully digital reporting workflows.
• Different supportive-supervision models.
• Community-level malaria reporting.
• Private-sector integration.
• Automated RDT readers.
• Interoperability between point-of-care tools and national HMIS platforms.
• Routine data-quality auditing and feedback systems.

Economic evaluations should include not only the initial purchase of devices or software but also connectivity, training, replacement, hosting, cybersecurity, maintenance, technical support, and the opportunity cost of healthcare workers' time. Policy priorities proposed in this review include maintaining a single national indicator dictionary, assigning responsibility for each transfer point, integrating private and community providers where feasible, and publishing routine data-quality findings [11], [13], [14]. These priorities are informed by the reviewed evidence but should not be interpreted as interventions proven effective in every setting.

## 9 Limitations

This review has several limitations. The available evidence was geographically concentrated and heterogeneous in study design, indicator definitions, and outcome measurement. Findings from selected countries cannot be assumed to represent the entire African continent. Some relevant operational evidence may be contained in unpublished programme reports and may not have been identified through the literature search. The available literature also provides limited evidence concerning the long-term sustainability, cost-effectiveness, privacy implications, and equity of digital and automated interventions. Finally, this review focuses principally on the post-test data pathway. It therefore does not provide a comprehensive assessment of the diagnostic performance of malaria RDTs.

## 10 Conclusion

Reliable malaria surveillance requires both a valid diagnostic result and an intact information pathway. Recording accuracy, aggregation accuracy, completeness, concordance, denominator availability, and timeliness should be measured separately because they arise at different stages and require different responses.

Standardized indicators, source-document verification, supportive supervision, and interoperable offline-capable systems may strengthen routine malaria data. Nevertheless, interventions should be adapted to local conditions and evaluated before large-scale implementation.

Every malaria RDT result should be considered the beginning of a traceable information pathway rather than merely the end of a diagnostic encounter. Strengthening that pathway could improve the credibility, interpretation, and practical usefulness of routine malaria surveillance.

## Author contribution

Abdullahi Hassan Elmi conceived the review, conducted the literature search, synthesized the evidence, drafted the manuscript, revised it critically for important intellectual content, and approved the final version.

## CRediT authorship contribution statement

**Abdullahi Hassan Elmi:** Writing – review & editing, Writing – original draft, Visualization, Validation, Supervision, Software, Resources, Project administration, Methodology, Investigation, Funding acquisition, Formal analysis, Data curation, Conceptualization.

## Funding

This Research Supported by SIMAD University.

## Declaration of competing interest

The author declares no competing interests.
